# Supplementary material for: Data quantity is more important than its spatial bias for predictive species distribution modelling
Source: PeerJ. 2020 Nov 27;8:e10411. doi: 10.7717/peerj.10411 (PMC7703440; doi:10.7717/peerj.10411)
Supplement: Figure S1 — Variables were chosen to represent a range of spatial patterns and scales of spatial auto-correlation. Units of measurement for variables are: degrees Celcius (minimum temperature and maximum temperature); millimeters (annual precipitation); hecto Pascals (atmospheric pressure); proportion of grid square covered by land cover (agricultural areas, artificial surfaces, forest & semi-natural, wetlands, water bodies); meters (elevation). [file peerj-08-10411-s001.pdf]

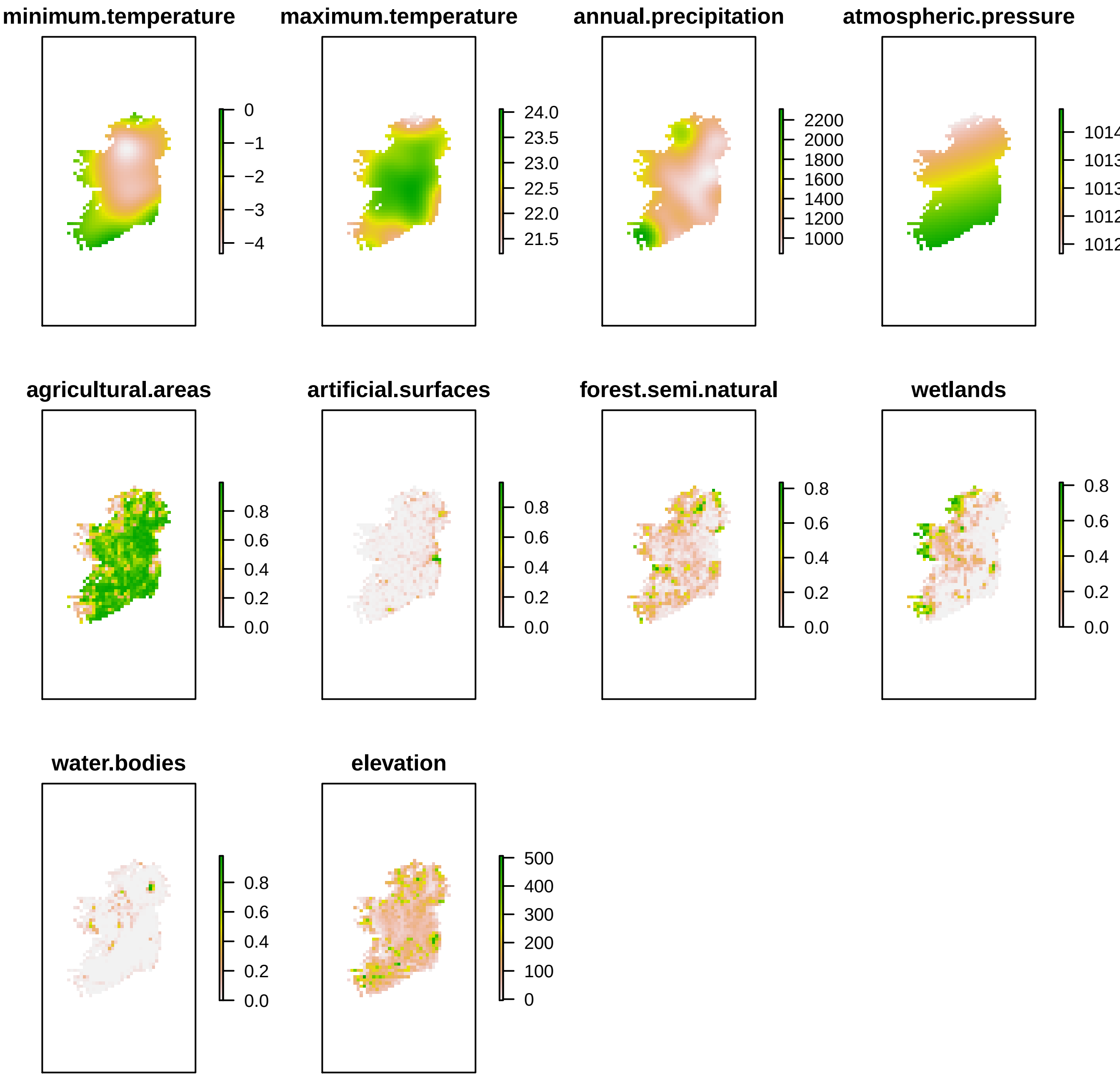

minimum.temperature
0
−1
−2
−3
−4
maximum.temperature
24.0
23.5
23.0
22.5
22.0
21.5
annual.precipitation
2200
2000
1800
1600
1400
1200
1000
atmospheric.pressure
1014
agricultural.areas
0.8
0.6
0.4
0.2
0.0
artificial.surfaces
0.8
0.6
0.4
0.2
0.0
forest.semi.natural
0.8
0.6
0.4
0.2
0.0
wetlands
0.8
0.6
0.4
0.2
0.0
water.bodies
0.8
0.6
0.4
0.2
0.0
elevation
500
400
300
200
100
0
