# Supplementary material for: Data quantity is more important than its spatial bias for predictive species distribution modelling
Source: PeerJ. 2020 Nov 27;8:e10411. doi: 10.7717/peerj.10411 (PMC7703440; doi:10.7717/peerj.10411)
Supplement: Figure S2 — We simulated an observation process that produces “presence only” records of a list of species at a location (A), which is a common format for biological records data. We then inferred species non-detections (represented as “0” in B) for every species in the community. This produced complete detection/non-detection data for every species on every checklist (B). We used the detection/non-detection data (B) to train species distribution models. [file peerj-08-10411-s003.pdf]

A

checklist ID	longitude	latitude	list length	species name
L14	75000	85000	4	sp2
L14	75000	85000	4	sp6
L14	75000	85000	4	sp5
L14	75000	85000	4	sp1

B

checklist ID	longitude	latitude	list length	sp 1	sp 2	sp 3	sp 4	sp 5	sp 6	sp 7	...	sp240
L14	75000	85000	4	1	1	0	0	1	1	0	...	0
