# Supplementary material for: Data quantity is more important than its spatial bias for predictive species distribution modelling
Source: PeerJ. 2020 Nov 27;8:e10411. doi: 10.7717/peerj.10411 (PMC7703440; doi:10.7717/peerj.10411)
Supplement: Table S1 — The full model (1st line) models AUC as a function of spatial bias and a smooth of sample size by spatial bias. The 2nd and 3rd lines show the the adjusted R2 of models with the sample size or spatial bias terms removed, respectively. Removing the sample size term reduces the adjusted R2 more than removing the spatial bias term does. [file peerj-08-10411-s006.docx]

**Table S1. Adjusted R^2^ of generalized additive models (GAMs) modeling prediction performance (AUC) of species distribution models for simulated species.** The full model (1^st^ line) models AUC as a function of spatial bias and a smooth of sample size by spatial bias. The 2^nd^ and 3^rd^ lines show the the adjusted R^2^ of models with the sample size or spatial bias terms removed, respectively. Removing the sample size term reduces the adjusted R^2^ more than removing the spatial bias term does.

| **Model** | **Adj. R^2^ (GLM SDMs)** | **Adj. R^2^ (inverse distance-weighted interpolation SDMs)** | **Adj. R^2^ (boosted regression tree SDMs)** |
| --- | --- | --- | --- |
| full model | 0.45 | 0.22 | 0.13 |
| - spatial bias | 0.43 | 0.21 | 0.11 |
| - sample size | 0.01 | 0.01 | 0.03 |
